# Study on the Coupling Coordination between Ecological Environment and High-Quality Economic Development in Urban Agglomerations in the Middle Reaches of the Yangtze River

**DOI:** 10.3390/ijerph20043612

**Published:** 2023-02-17

**Authors:** Ying Zhang, Zhiqiang Fang, Zhongqi Xie

**Affiliations:** 1 School of Humanities and Law, Yanshan University, Qinhuangdao 066004, China; 2 Yanshan University Press, Yanshan University, Qinhuangdao 066004, China

**Keywords:** ecological environment, high-quality economic development, coupling coordination model

## Abstract

The ecological environment is the basis of high-quality economic development, and the coordinated development of the two is of great significance for promoting regional sustainable development. This study takes 31 cities in the middle reaches of the Yangtze River as samples, constructs an evaluation index system of the ecological environment (EE) and high-quality economic development (HQED), and uses a comprehensive evaluation method and a coupling coordination degree model to determine the development level, coupling coordination relationship, interaction coordination relationship and space–time evolution characteristics of the two. The results show that: (1) In the sample period, the overall level of EE and HQED increased simultaneously, but the two indexes of each city showed obvious heterogeneity. (2) EE and HQED have a coupling coordination relationship; the coupling degree (CD) is in a high coupling stage, and the coupling coordination degree (CCD) is in a good and moderate coordination state. (3) In the interactive coordination relationship, the CCD sequence of subsystems is coordinated development > shared development > innovative development > open development, and pressure subsystem > response subsystem > status subsystem. This study provides a new evaluation perspective for EE and HQED and puts forward suggestions for their coupling and coordinated development.

## 1. Introduction

With the excessive consumption of resources and the continuous pollution of the environment, the contradiction between global economic development and the ecological environment is intensifying [1]. Ecological environment (EE) protection and high-quality economic development (HQED) are important conditions for achieving regional sustainable development. China’s economy is facing the urgent need for high-quality development. From the perspective of per capita GDP, China’s per capita GDP will reach USD 10,500 in 2020, still lower than the USD 10,926 global per capita GDP. China is still a big developing country, and its economy is at a stage of high-speed growth toward high-quality development. China’s 14th Five-Year Plan emphasizes “promoting high-quality development”. Since it was published, HQED has become China’s long-term strategic goal [2].

As far as EE evaluation research is concerned, there are not only the measurements of existing biological resources [3], but also research on environmental health and ecosystem vitality with drinking water safety, air quality, pollution discharge and other evaluation indicators [4]. The evaluation research on the quality of economic development includes building a comprehensive index system based on different spatial scales [5] and building an evaluation system from a certain dimension or index [6], as well as structural evaluation for the internal economy [7]. HQED evaluation and economic development evaluation focus on different evaluation dimensions, including and expanding the content of the original economic evaluation and paying more attention to sustainability, coordination, efficiency and fairness [8]. HQED will be China’s goal for a long period of time into the future and is of great concern to Chinese scholars. It is a high-quality development concept with Chinese characteristics and has been widely used in economic [9], education [10], tourism [11] and other related fields. With regard to the theoretical interpretation of the HQED concept, Ren et al. believes that high-quality development should be measured by coordination, innovation, sustainability, sharing and effectiveness [12]. Han et al. believes that high-quality development is to achieve effectiveness in the economic and social aspects, and to innovate various social constructions from the perspective of coordinated development [13].

As far as the coupling and coordination research of the two is concerned, EE evaluation research has always been in a subordinate position to economic development research, rather than there being a mutual relationship between coupling and coordinated development [14]. Simon Kuznets put forward the Environmental Kuznets Curve (EKC) to describe the relationship between EE and economic development [15]. Many scholars have studied the income level and environmental quality of different countries and regions, and found various relationships such as U shape, M shape, N shape, etc. [16,17]. In addition, some scholars have tried to explore the coupling relationship between the two, such as the evolution of the integration of environmental protection and high-quality development of the industrial economy [18], and the study of urban economic development [19]. There has also been research on the coupling of economic growth and carbon emissions, etc. [20].

According to existing literature, the academic research on EE and HQED still has the following deficiencies: first, rich research results have been achieved on HQED, but the comprehensive evaluation and evaluation index system of EE and HQED are still insufficient. Second, there is only a small amount of literature on the coupling and coordination of EE and HQED. EE is the primary bottleneck constraining economic development [21] and is also the key factor for promoting HQED [22]. Therefore, the necessity of coupling and coordinated development of the two should be studied and discussed holistically in order to foster sustainable development of the regional economy. Specifically, in order to make up for the lack of existing research, this paper tries to solve the following three problems: (1) How to build an evaluation index system of EE and HQED and evaluate the comprehensive development level. (2) Determining whether there is a coupling coordination relationship between EE and HQED. (3) Identifying what kind of coupling and coordination relationship types and space–time characteristics exist in the sample space.

**Figure 1:**
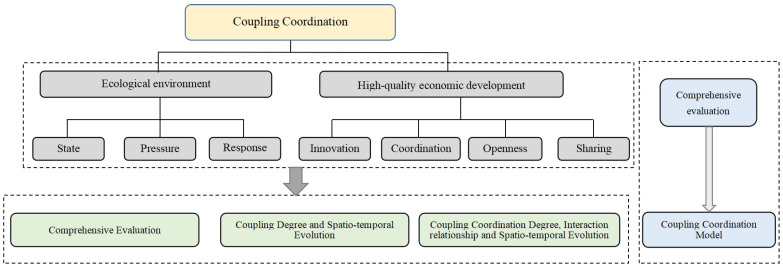
Research design.

Based on the measurement of the EE and HQED levels of 31 cities in the urban agglomeration of the middle reaches of the Yangtze River in China from 2010 to 2019, this study examines the comprehensive development level, coupling and coordination relationship, and space–time evolution characteristics of EE and HQED. Compared with the existing research, the contributions of this study are as follows: (1) Based on the PSR model and the concept of high-quality development, a comprehensive evaluation index system of EE and HQED is built. (2) The urban agglomeration in the middle reaches of the Yangtze River is taken as the sample space, and the coupling coordination type, interaction coordination relationship and space–time evolution characteristics of EE and HQED are measured and evaluated. In particular, the analysis of the interaction and coordination relationship further explores the key factors affecting the coordinated development (3). This paper points out the problems and weaknesses of EE and HQED in the urban agglomeration in the middle reaches of the Yangtze River from a practical perspective and puts forward countermeasures and suggestions to promote the coupling and coordinated development of the two. The design and flow of this study are shown in Figure 1.

## 2. Methodology and Materials

### 2.1. Study Area

**Figure 2:**
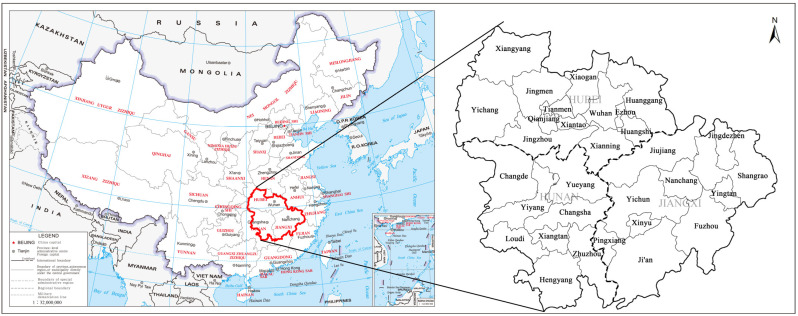
Urban agglomerations in the middle reaches of the Yangtze River.

The urban agglomeration in the middle reaches of the Yangtze River is located in central China. It covers 31 cities and an area of 326,100 square kilometers spanning the Hubei, Hunan and Jiangxi provinces, and is the largest urban agglomeration in China (Figure 2) [23]. According to the China Statistical Yearbook, in 2020, the permanent population within the urban agglomeration in the middle reaches of the Yangtze River will account for about 9.4% of the country, and the GDP will account for about 9.3% of the country. The urban agglomeration in the middle reaches of the Yangtze River has unique geographical conditions. It faces the river and the sea and connects the east and the west, as well as the north and the south. It has a vast economic hinterland. It is in an important strategic position in China’s construction of a pattern of major domestic circulation and dual domestic and international circulation [24]. Figure 2 is from the Ministry of Natural Resources of China, drawing review No. GS (2019) 1679.

### 2.2. Data Sources and Indicators

The data involved in this study include the China Regional Economic Statistical Yearbook (2010–2014), the China Statistical Yearbook (2011–2020), the Hubei Statistical Yearbook (2011–2020), the Hunan Statistical Yearbook (2011–2020), and the Jiangxi Statistical Yearbook (2011–2020).

The evaluation index system is the foundation to study the coupling and coordinated development of EE and HQED [25]. Based on the systematic review of relevant literature, this research builds an indicator system to comprehensively evaluate EE and HQED through comparative analysis and expert discussion. In terms of EE indicators, based on causal logic [26], this study deploys the PSR model (stress, state, response) to design the content of EE indicators. This is a common model for environmental quality assessment and is widely used in the design of EE-related indicators [27]. It complies with the index design principles of operability, representativeness and scientificity. Among them, the status subsystem includes the per capita green area and the green coverage rate of the built-up area. The pressure subsystem includes smoke (powder) dust emission per unit of industrial added value and exhaust gas emission per unit of industrial added value. The response subsystem includes domestic waste treatment rate and sewage treatment rate. Furthermore, compared with existing studies, some parameters have not been included in the indicator system, such as the per capita total grain [28], per capita industrial sulfur dioxide emissions [29], etc., due to the impact of changes in statistical indicators, data classification adjustment, statistical continuity and other factors.

HQED indicators are combined with the concept of high-quality development to build the HQED evaluation index system, using indicators from the four subsystems of innovative development, coordinated development, open development and shared development (Table 1). Different from previous studies that focus on the quantitative characteristics of economic development [30], the evaluation dimension of HQED can more comprehensively reflect the sustainability [31], fairness [32] and stability [33] of economic development. Among them, innovation is the driving force of HQED. From the perspectives of innovation investment and innovation contribution, three indicators are selected to measure the innovative development level of the economy: the proportion of scientific research personnel expenditure in GDP, the number of scientific research personnel per 10,000 people, and the proportion of high-tech enterprises in the country. Coordination is the inherent requirement of high-quality development. Three indicators are selected to measure the level of coordinated development: the per capita GDP reflects the level of income coordination, the ratio of per capita consumption level to the national consumption level reflects the level of consumption coordination, and the proportion of the output value of the secondary and tertiary industries to the total output value reflects the characteristics of the industrial structure. Openness is the condition for the realization of HQED, and it is also the key link for the realization of HQED in the urban agglomeration in the middle reaches of the Yangtze River. The three indicators of foreign investment, foreign trade and tourism are selected to measure openness. Sharing is the fundamental pursuit of high-quality development. Three indicators are selected, including the number of doctors per thousand people, the proportion of social security and employment expenditure in general public budget expenditure, and the registered unemployment rate, which specifically reflect the social welfare, social security and social stability.

### 2.3. Research Method

#### 2.3.1. Entropy Weight Method and Comprehensive Evaluation index

(1) Standardized processing.

(1) $$A_{ij}=\frac{X_{ij}-\min(X_{ij})}{\max(X_{ij})-\min(X_{ij})}$$

(2) $$A_{ij}=\frac{\max(X_{ij})-X_{ij}}{\max(X_{ij})-\min(X_{ij})}$$

(2) Index weight calculation.

(3) $$P_{ij}=\frac{A_{ij}}{\sum_{j=1}^{n}A_{ij}},W_{i}=\ln\frac{1}{n}\sum_{j=1}^{n}\left[\left(P_{ij}\right)\ln\left(P_{ij}\right)\right]$$

(4) $$\lambda_{ij}=\frac{\left(1-W_{i}\right)}{\sum_{i=1}^{m}\left(1-W_{i}\right)}$$

(3) Calculation of comprehensive evaluation index.

(5) $$u_{1}=\sum_{i=1}^{m}\lambda_{ij}A_{ij},u_{2}=\sum_{i=1}^{m}\lambda_{ij}B_{ij},\;\sum_{i=1}^{m}\lambda_{ij}=1$$

#### 2.3.2. Coupling Coordination Model

The coupling coordination model is used to analyze the coordinated development level of various factors [47]. In this study, coupling degree (CD) is used to reflect the degree of interaction between EE and HQED, and coupling coordination degree (CCD) is used to reflect the degree of coordination, especially the degree of benign coupling when the two interact.

(6) $$C=\frac{\sqrt{\left(u_{1}\times u_{2}\right)}}{\left(u_{1}+u_{2}\right)},T\left(u_{1},u_{2}\right)=au_{1}+bu_{2}$$

(7) $$D\left(u_{1},u_{2}\right)=\sqrt{C\left(u_{1},u_{2}\right)T\left(u_{1},u_{2}\right)}$$

## 3. Results

### 3.1. Comprehensive Index Analysis 

**Figure 3:**
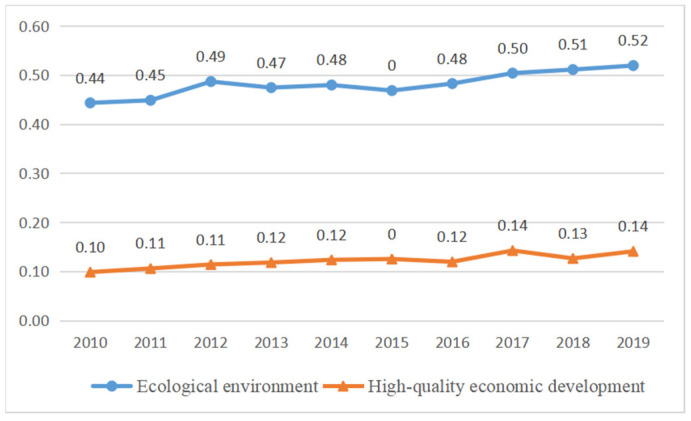
Trend chart of comprehensive evaluation index of EE and HQED in 2010–2019.

**Figure 4:**
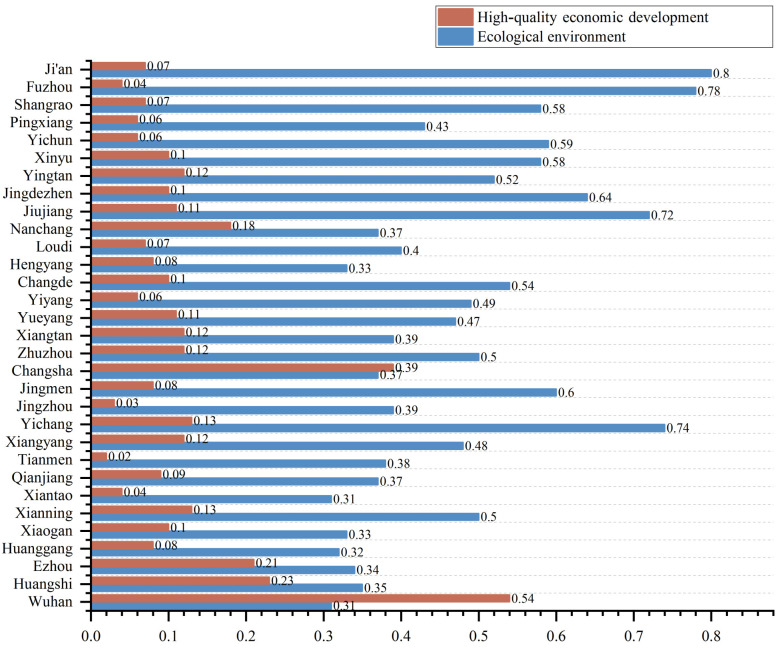
Comprehensive evaluation index of 31 cities’ EE and HQED.

**Figure 5:**
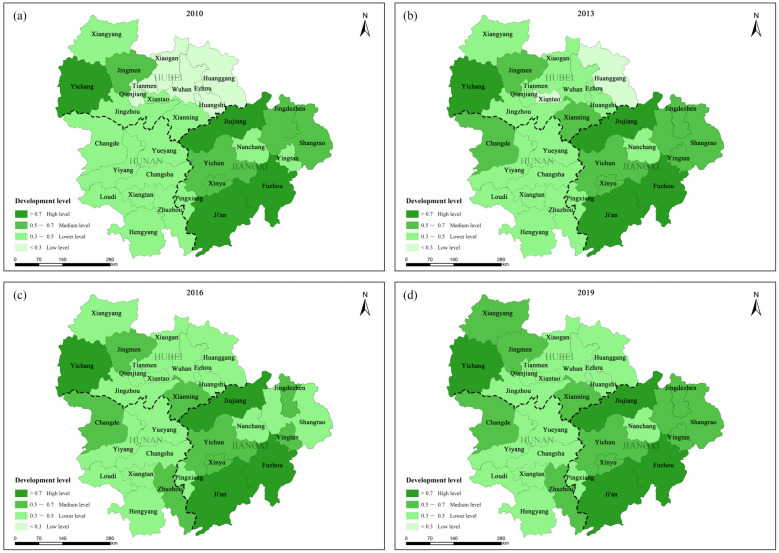
Temporal and spatial changes of the comprehensive assessment level of EE from: (**a**) 2010; (**b**) 2013; (**c**) 2016; (**d**) 2019.

(1) From the perspective of a time series, the EE composite index and HQED composite index of the urban agglomeration in the middle reaches of the Yangtze River show a synchronous growth trend. With economic development, the EE has continuously improved. As shown in Figure 3, the EE composite index of the urban agglomeration in the middle reaches of the Yangtze River rose from 0.44 in 2010 to 0.52 in 2019. The HQED composite index rose from 0.10 in 2010 to 0.14 in 2019. Thus, the EE and HQED of the urban agglomeration in the middle reaches of the Yangtze River show a good trend. 

(2) By calculating the average value of the EE index and HQED index of 31 cities from 2010 to 2019, and from the average value of the EE and HQED composite index of each city(see Figure 4), it was found that the EE index and HQED index of each city were significantly different. Of the 31 cities, only Wuhan and Changsha had a higher HQED index than EE index; of the two, Wuhan’s HQED index (0.54) was significantly higher than the EE index (0.31) while Changsha’s HQED index (0.39) was basically the same as the EE index (0.37). The EE and HQED of Ezhou and Huangshi were not much different. The HQED index of the other 27 cities was significantly lower than the EE index, especially for Ji’an, whose EE index was as high as 0.80, while the HQED index was only 0.07. The Fuzhou EE index was 0.78, significantly higher than its HQED index (0.04). For a long time, the emissions of pollutants from the economic development of cities have had adverse effects on EE, especially in heavy industrial cities such as Wuhan, where the development of industry has incurred serious environmental pollution problems [48]. In Ji’an, Fuzhou, Shangrao and other cities, the HQED index is at a low level among the 31 cities, and the EE index is at a high level. This phenomenon reflects that under a relatively low level of economic development, human damage to EE is relatively small [49].

(3) From the perspective of spatial distribution, the overall level of EE has been continuously improved, and it can be divided into Jiangxi > Hubei > Hunan by province (2019). In order to show the spatial difference, the EE index was classified into high level (>0.7), medium level (0.5–0.7), low level (0.3–0.5) and very low level (<0.3). At the same time, the EE index of 2010, 2013, 2016 and 2019 was determined to draw the space–time change diagram of the EE comprehensive level (Figure 5). Specifically, Ji’an, Fuzhou, Jiujiang and Yichang were at a high level in the four time-section datasets. The number of medium-sized cities is increasing. In 2010, only five cities, Jingdezhen, Shangrao, Xinyu, Yichun and Jingmen, were at the middle level. By 2013, there were eight medium-sized cities, and by 2019, there were 10 medium-sized cities. The number of lower-level cities increased from 15 in 2010 to 17 in 2019. In 2010, there were seven cities classified as low-level cities (Wuhan, Xiaogan, Tianmen, Qianjiang, Ezhou, Huanggang and Huangshi), all clustered around Wuhan. By 2013 and 2019, the EE level of these seven cities had risen, and all of them had been elevated to a classification of lower-level cities. The EE quality of Jiangxi Province has always been in the leading position in the central region. It has issued the guidance and action plan for the construction of ecological civilization, carried out a waste gas mine restoration project, and implemented wetland biodiversity protection, migratory bird habitat restoration, water ecological restoration and other protection and restoration projects focusing on the Poyang Lake basin [50].

**Figure 6:**
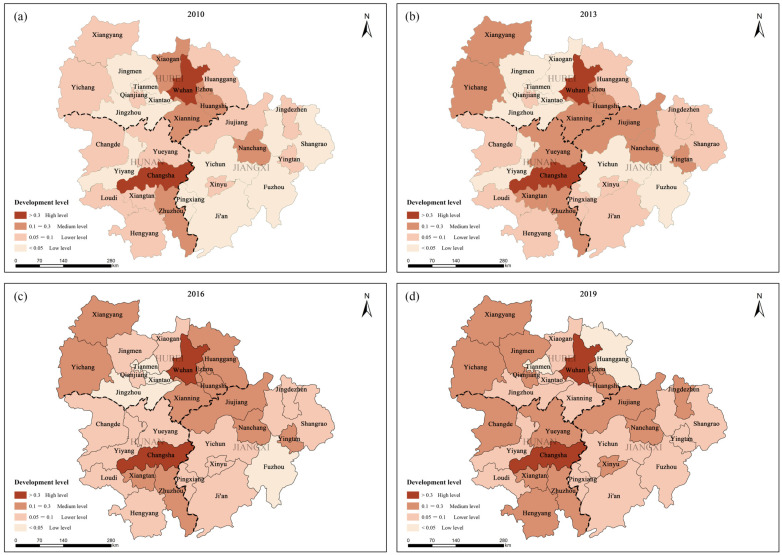
The space–time changes of the comprehensive evaluation level of HQED from: (**a**) 2010; (**b**) 2013; (**c**) 2016; (**d**) 2019.

Regarding the spatial distribution of the HQED level, the following order was observed: Hunan > Hubei > Jiangxi. The HQED level was classified into high level (>0.3), medium level (0.1–0.3), low level (0.05–0.1) and very low level (<0.05), and the HQED indexes of 2010, 2013, 2016 and 2019 were determined to draw the comprehensive level space–time change map (Figure 6). In general, Wuhan and Changsha were always at a high level. The number of medium-sized cities has been increasing, from six cities in 2010 (Huangshi, Xiaogan, Ezhou, Nanchang, Xianning, Zhuzhou) to fifteen cities in 2019 (Xianning removed, Yichang, Qianjiang, Jiujiang, Xiangyang, Yueyang, Jingdezhen, Changde, Jingmen, Hengyang, Xinyu added). Low-level cities have been decreasing, from 10 in 2010 to only two in 2019 (Huanggang and Tianmen). Huanggang belongs to the Dabie Mountains, with a weak economic foundation and a relatively good agricultural foundation. Tianmen is located in the Hanjiang Plain, with rich fishery and agricultural resources, and relatively weak secondary and tertiary industries. Therefore, the HQED level of the two places was lowered. Wuhan and Changsha are the provincial capitals of Hubei and Hunan, and they are also the core cities that drive the development of the urban agglomeration in the middle reaches of the Yangtze River. Relying on the original industrial base and convenient transportation location, they have been at the forefront of the economic development of the urban agglomeration in the middle reaches of the Yangtze River [51,52]. In recent years, the two cities have continuously strengthened industrial resources and technological innovation. For example, relying on the original automobile industry, Wuhan has focused on building new energy vehicles, cooperating with Changsha and Zhuzhou to promote the transformation of electric vehicles, and driving the Nanchang metropolitan area to develop new energy vehicles to maintain the advantages of special vehicles. At the same time, it has helped Yichun, Changde, Hengyang, Jiujiang, Jingzhou and other cities to build auto parts bases, promoted industrial cooperation within the urban agglomeration in the middle reaches of the Yangtze River, and optimized the regional layout of the industrial chain [53].

### 3.2. The Space–Time Evolution of CD

From the perspective of a time series, the CD changes of EE and HQED in the 31 cities over the past 10 years were mapped and the CD was graded, as shown in Table 2 and Table 3. Overall, the CD of most cities in the urban agglomeration in the middle reaches of the Yangtze River was more than 0.7, which is at the advanced coupling level. In 2019, the proportion of cities with an advanced coupling level reached 84%, indicating that the EE and HQED of most cities in the urban agglomeration in the middle reaches of the Yangtze River formed a coupling relationship.

**Figure 7:**
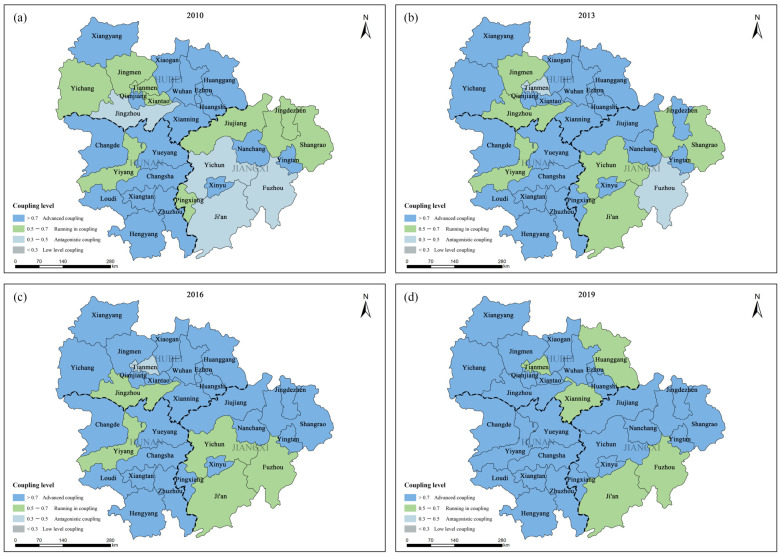
Spatial distribution of coupling level of 31 cities in: (**a**) 2010; (**b**) 2013; (**c**) 2016; (**d**) 2019.

From the perspective of spatial distribution, the overall coupling level of EE and HQED in the urban agglomeration in the middle reaches of the Yangtze River has been continuously improved, with Hunan > Hubei > Jiangxi. In order to more accurately reflect the spatiotemporal changes in CD of the 31 cities in the urban agglomeration in the middle reaches of the Yangtze River, the CDs of 2010, 2013, 2016 and 2019 were determined for mapping (Figure 7). It can be clearly seen that Wuhan, Changsha and Nanchang, the central cities of the urban agglomeration in the middle reaches of the Yangtze River, were always in the advanced coupling stage. The running-in coupling was reduced and upgraded to advanced coupling for cities such as Yichang, Jingmen, Yiyang, Jiujiang, Shangrao, Jingzhou and Pingxiang. Antagonistic coupling decreased from four in 2010 to zero in 2019. In general, with the economic development and the deepening of the integration of the urban agglomeration in the middle reaches of the Yangtze River, the CD of EE and HQED also increased. In particular, Wuhan, Changsha and Nanchang, as the provincial capital cities, have always been in the advanced coupling stage due to their high level of economic development, relatively complete ecological protection measures, and the coupling relationship between EE and economic development that is mutually promoting, interdependent and closely coordinated.

### 3.3. Spatiotemporal Evolution of CCD

#### 3.3.1. The Coupling Coordination Relationship between EE and HQED

From the perspective of a time series, the CCD of EE and HQED in the 31 cities has improved continuously in the past 10 years, but it is still only at or below a good coordination level, and there is no city with high-quality coordination. CCD is classified as shown in Table 4 and Table 5. The CCD level mainly includes high-quality coordination, good coordination, moderate coordination and low coordination.

**Figure 8:**
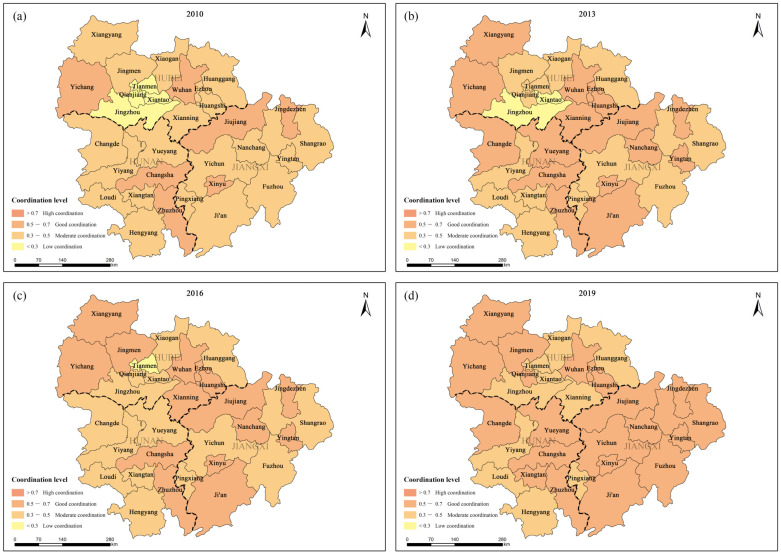
Spatial distribution of coupling coordination level of 31 cities in: (**a**) 2010; (**b**) 2013; (**c**) 2016; (**d**) 2019.

From the perspective of a spatial distribution, the overall coordination level of EE and HQED in the urban agglomeration in the middle reaches of the Yangtze River is constantly improving, with Jiangxi > Hunan > Hubei. The CCD images of the 31 cities from 2010, 2013, 2016 and 2019 were captured for mapping (Figure 8). From the spatiotemporal changes of these CCDs, it can be clearly seen that the urban agglomeration in the middle reaches of the Yangtze River is mainly at the two coordination levels; namely, good coordination and moderate coordination. In 2019, there were 21 good-coordinated cities (accounting for 68%) and 10 moderately coordinated cities (accounting for 32%). Specifically, in 2010, the CCD of Tianmen, Xiantao, Qianjiang and Jingzhou cities was lower than 0.3, i.e., at the low-coordination level. In 2013, the number of low-coordination areas decreased by two, leaving only Xiantao (0.293) and Jingmen (0.288). In 2016, low-coordination areas continued to decrease, leaving only Tianmen (0.294). In 2019, there were no low-coordination cities, and other cities in Jiangxi, except for Pingxiang, rose to a good-coordination level. Especially in Jiangxi Province, the CCD level of EE and HQED improved as a whole. Jiangxi Province is a major agricultural province in China with rich ecological resources and a good environmental foundation [54]. In recent years, Jiangxi has developed innovative infrastructure relying on the Poyang Lake Innovation Demonstration Zone and the science and technology innovation corridor on both sides of the Ganjiang River. It has development advantages in the national backbone of the internet direct connection points and new energy special vehicles, which has significantly improved the CCD level of EE and HQED.

#### 3.3.2. Coupling Coordination Relationship between EE and the HQED Subsystem

**Figure 9:**
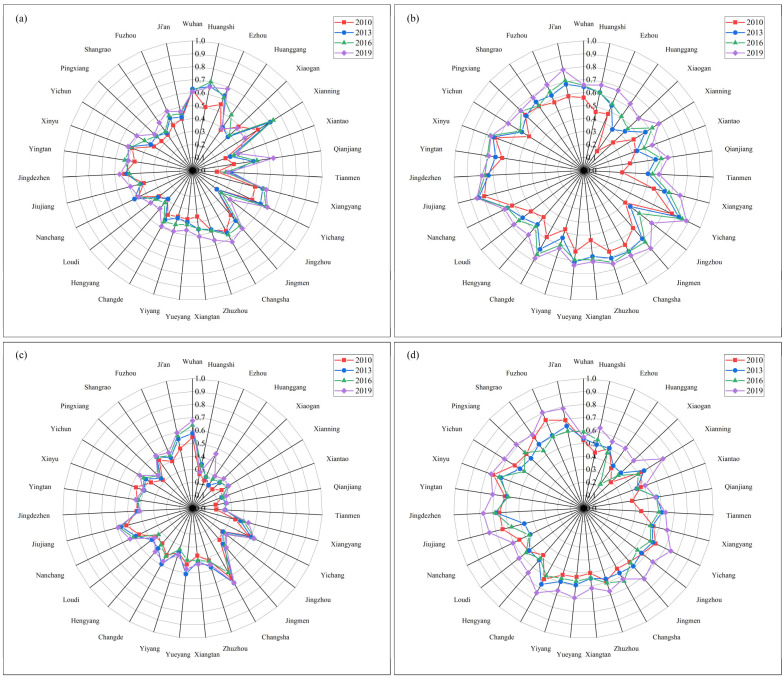
CCD of EE and the HQED subsystem: (**a**) The CCD of EE and the innovative development subsystem; (**b**) The CCD of EE and the coordinated development subsystem; (**c**) The CCD of EE and the open development subsystem; (**d**) The CCD of EE and the shared development subsystem.

The coupling coordination relationship between EE and the four subsystems of HQED is shown in Figure 9. From the overall level of CCD, the coupling coordination level of the four subsystems of EE and HQED shows coordinated development > shared development > innovative development > open development. (1) From the perspective of coordination with the coordinated development subsystem, in 2019, the EE of 14 cities, (including Xiangyang, Yichang, Jingmen and Changsha) and the CCD of the coordinated development subsystem were at a high-quality coordination. The remaining 17 cities were at a good coordination level. (2) From the perspective of coordination with the shared development subsystem, the EE of 12 cities such as Xianning, Yichang, Jingmen and Changde were at a high-quality coordination with the CCD of the shared development subsystem, while the other 19 cities were at a good level of coordination. (3) From the perspective of coordination with the innovation and development subsystem, only Xianning’s CCD reached a high level of coordination in 2013 and 2016. In 2019, the CCD of 13 cities including Wuhan, Huangshi, Ezhou and Qianjiang reached a good-coordination level, while the other 18 cities were at a moderate-coordination level. (4) From the perspective of coordination with the open development subsystem, Hunan > Jiangxi > Hubei. Among them, Huangshi, Xiantao, Qianjiang and Tianmen were at a low level of coordination. Wuhan, Yichang, Changsha, Nanchang, Jiujiang and Ji’an were at a good level of coordination, while the other 21 cities were at a moderate level of coordination. In the 2019 GDP ranking of Hubei Province, Huangshi, Xiantao, Qianjiang and Tianmen ranked low [55], while the industrial base was relatively weak and the degree of opening up was relatively low. The radiation and driving role of Wuhan, the central city, should be utilized to strengthen the development of foreign investment, foreign trade and tourism industry.

#### 3.3.3. Coupling Coordination Relationship between HQED and the EE Subsystem

**Figure 10:**
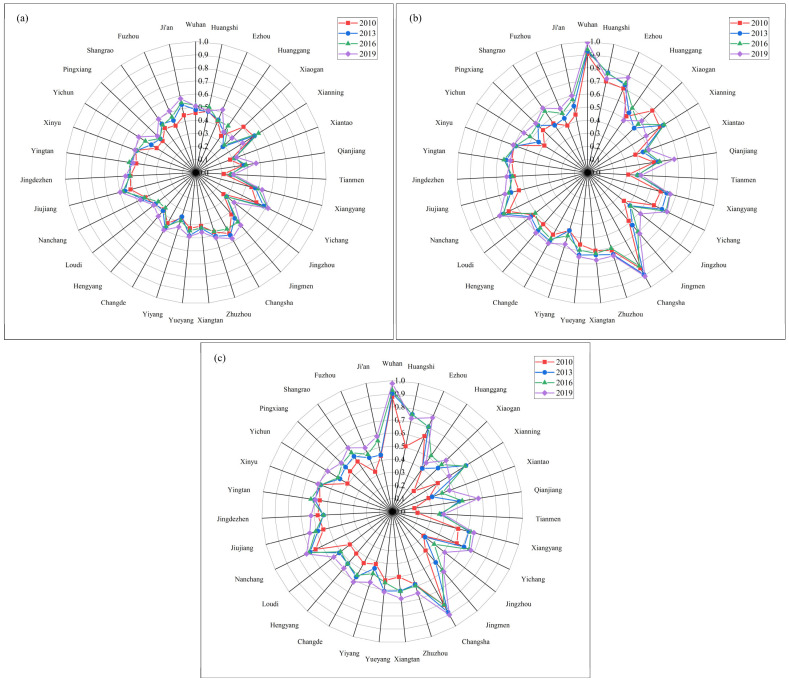
CCD of HQED and the EE subsystem: (**a**) CCD of HQED and the status subsystem; (**b**) HQED and CCD of the pressure subsystem; (**c**) HQED and CCD of the response subsystem.

The coupling coordination relationship between HQED and the three subsystems of EE is shown in Figure 10. From the overall level of CCD, the CCD of the HQED and three subsystems show pressure subsystem > response subsystem > state subsystem (1). From the perspective of coordination with the pressure subsystem, the EE and the CCD of the response subsystem in Wuhan (0.994), Changsha (0.906), Ezhou (0.789), Nanchang (0.748) and Huangshi (0.731) were at a high-quality coordination in 2019. Huanggang (0.485) and Tianmen (0.411) were at a moderate level of coordination. The remaining 24 cities were at a good level of coordination. (2) According to the coordination relationship with the response subsystem, Wuhan (0.978), Changsha (0.901), Ezhou (0.780), Nanchang (0.732) and Huangshi (0.726) were at a high-quality coordination. Xiantao (0.467), Huanggang (0.454) and Tianmen (0.395) were at a moderate level of coordination. The remaining 23 cities were at a good level of coordination. (3) From the coordination relationship with the state subsystem, there were only two coordination levels: moderate coordination (18 cities) and good coordination (13 cities). It can be seen from the indicator system that the EE status subsystem was relatively stable but also vulnerable to damage. As shown in Figure 10a, the CCD of EE and status subsystems of cities in the middle reaches of the Yangtze River urban agglomeration were relatively stable and the gap was relatively small, indicating that the impact on EE status was relatively small in the development of the middle reaches of the Yangtze River urban agglomeration. At the same time, the CCD of the state subsystem and HQED was also relatively low. The relationship between the pressure subsystem and the response subsystem and the level of economic development is relatively close. It can be clearly seen from the radar chart that the higher the HQED level of cities such as Wuhan and Changsha, the higher the CCD of their HQED and the pressure subsystem and response subsystem.

## 4. Discussion 

### 4.1. Discussion and Recommendations

In recent years, the regional economy connected by urban agglomeration has developed rapidly, and the rapid economic development has exerted great pressure on the ecological environment [56]. The urban agglomeration in the middle reaches of the Yangtze River is the emerging growth pole of China’s economy [57]. At the same time, the ecosystem is complex and fragile. The coordination and co-construction of EE and HQED has become the primary issue for regional economic sustainable development. The existing research mainly focuses on the relationship between EE and urbanization. For example, Wu et al. examined the coupling relationship between urbanization and EE in Harbin Great Wall City Group; they observed that population urbanization has transformed into social urbanization and that the relationship with EE depicts a U-shaped curve [28]. Zou et al. evaluated the coupling coordination relationship and spatial heterogeneity characteristics of urbanization and EE in Shaanxi Province, and learnt that the development of ecological environment is lower than the speed of development of urbanization [29]. The coupling research on EE and HQED is relatively small. HQED is the inevitable choice of China’s economy, moving from focusing on quantity to focusing on quality. Therefore, exploring the coupling and coordination relationship between EE and HQED was the focus of this study, with the hope of providing support for high-quality development and sustainable development of China’s regional economy.

According to the evaluation results of the comprehensive evaluation index system of EE and HQED, the EE index and HQED index of the urban agglomeration in the middle reaches of the Yangtze River showed a synchronous growth trend during the sample period. However, from the perspective of the average value of the index of the 31 cities, the EE index and HQED index of all cities, with the exception of Changsha, showed significant differences. For example, the EE index of Ji’an and Fuzhou ranked first and second among the 31 cities, but the HQED index only ranked 23rd and 28th. Wuhan ranked first in the HQED index but 31st in the EE index. This feature conforms to the inverted U-shaped curve of environmental Kuznets [58], but the inflection point has not yet appeared. Economic growth and EE can be balanced, but the prerequisite is to implement effective environmental policies while ensuring economic growth. Therefore, environmental policies should be issued and implemented in a timely manner to promote the simultaneous development of the two. For example, Changsha has implemented the Xiangjiang River protection and treatment plan for many years, promoted the comprehensive treatment project of rivers, lakes and rivers, and strictly implemented the supervision and law enforcement work. EE and HQED have achieved synchronous development in the 31 cities.

From the analysis of the spatiotemporal evolution of the overall CD, CCD and interactive coordination relationship, it was found that EE and HQED have a significant coupling relationship and are at an advanced coupling level, but EE and HQED have not reached high-quality coordination, being always at the good and moderate levels of coordination during the sample period. Therefore, continuous attention should be paid to the improvement of the coordination level. Through the analysis of interaction and coordination, it was concluded that the innovative development and open development in the HQED subsystem, and the status subsystem in the EE subsystem, were the main factors that restricted the CCD of EE and HQED of urban agglomeration in the middle reaches of the Yangtze River. Coordinated development is the key proposition of regional economic development. Through CCD analysis, it was found that the coordinated development level of EE and HQED in the urban agglomeration in the middle reaches of the Yangtze River was low. For a long time, the urban agglomeration in the middle reaches of the Yangtze River has been one of the oldest and strongest industrial bases in China, relying on convenient land and water transportation and rich mineral resources, and has formed a biased industrial structure dominated by steel, building materials and automobiles. At the same time, there is the reality that the foundation of the tertiary industry is weak and the industrial technology is relatively backward, resulting in a low level of innovation development and an open development subsystem. This urban agglomeration is located in the middle reaches of the golden waterway of the Yangtze River, and is the key area for ecological protection of the Yangtze River Economic Belt. At present, the state subsystem of EE in the urban agglomeration in the middle reaches of the Yangtze River also lowers the coordination relationship.

Based on the above research, this paper puts forward the following policy recommendations.

First, adhere to the principle of ecological priority. Good EE is the fairest public product. Since China’s reform and opening, rapid economic development has been at the cost of environmental pollution. EE is the inherent requirement of sustainable development of human civilization [59]. With an increasing income level, people’s demand for high-level EE is increasingly strong. At the same time, the decline of human civilization is often caused by climate change, soil desertification and other factors [28]. The coordination between EE and HQED directly affects the ecosystem of the Yangtze River. Therefore, in view of the fact that EE lags behind in the development of HQED in certain regions, the principle of ecological priority should be followed.

Second, establish an ecological economic system. Chairman Jinping Xi proposed that lucid waters and lush mountains are invaluable assets. This concept highlights the economic value of ecology. High-level EE is conducive to gathering various economic resources, production factors and high-end talents, thus facilitating production development and technological innovation. The urban agglomeration in the middle reaches of the Yangtze River can rely on scientific and technological innovation to build a green technology system and expand the energy conservation and environmental protection industry and clean energy industry. At the same time, relying on the advantages of EE, ecological industries, ecological agriculture, ecological tourism and other ecological economies should be developed so that the green resources of the urban agglomeration in the middle reaches of the Yangtze River can be transformed into ecological products and services. The establishment of an ecological economic system can compensate for the lack of coordinated development and strengthen the coordination and cooperation between EE and various industries through the overarching and systematic characteristics of the system.

Third, improve the evaluation system and institutional arrangement of HQED, and highlight the economic value of EE. The evaluation system is a baton, which integrates ecological benefits, resource consumption, environmental pollution and other indicators into an economic development evaluation system. It also integrates EE cost into the economic operation cost and establishes the pay-to-use system and ecological compensation system of intergenerational compensation resources, which is conducive to the rational use of ecological resources in the urban agglomeration in the middle reaches of the Yangtze River, and realizes the coupling and coordinated development of EE and HQED. At the same time, a perfect evaluation method can form horizontal comparisons and benign competition among cities, which is conducive to mutual learning among cities, and jointly achieving the coupling development of EE and HQED.

### 4.2. Limitations and Future Research Directions

Limited by the availability and continuity of data, some factors have not been included in the indicator system, resulting in certain limitations of the results. For example, the per capita total grain output of the status subsystem and the per capita industrial sulfur dioxide emissions of the pressure subsystem in the ecological indicators were not incorporated in the indicator system. However, if the time range of sample indicators can be expanded, the study will more clearly represent the coupling and coordinated evolution of EE and HQED in urban agglomeration in the middle reaches of the Yangtze River. Future research will further improve the coverage of the indicator system and expand the time range of the sample period.

## 5. Conclusions

EE and HQED restrict and promote each other. It is of great significance to study the coupling and coordination relationship between them for HQED and sustainable development. This study constructs a comprehensive evaluation index system of EE and HQED and measures the comprehensive level, CD, CCD, interaction and coordination of EE and HQED in the urban agglomeration in the middle reaches of the Yangtze River from 2010 to 2019, as well as the spatiotemporal change rules based on the evaluation results. These measurement results reveal that the EE and HQED of the urban agglomeration in the middle reaches of the Yangtze River have the following significant characteristics:

(1) From 2010 to 2019, the overall levels of EE and HQED in the urban agglomeration in the middle reaches of the Yangtze River were improved simultaneously; however, there was significant heterogeneity in specific cities.
(2) From 2010 to 2019, the EE and HQED of the urban agglomeration in the middle reaches of the Yangtze River had an advanced coupling relationship and a good/moderate-coordination level relationship. Therefore, improving the coordination between the two should be the focus in the future.
(3) Through the analysis of the interaction and coordination relationship, it was found that the CCD sequence of the four subsystems of HQED was coordinated development > shared development > innovative development > open development, and the CCD sequence of the three subsystems of EE was pressure subsystem > response subsystem > state subsystem.

## Figures and Tables

**Table 1:** Evaluation index system of EE and HQED.

System	Subsystem	Primary Indicators/Effect Direction (+/−)	Index Meaning	Weight	References
EE	State	The per capita green area (m^2^) (+)	Environmental state	0.6406	[29]
		Green coverage rate of built-up area (%) (+)	Environmental state	0.0788	[29]
	Pressure	Smoke (powder) dust emission per unit industrial added value (10,000 ton) (−)	Environmental pollution	0.0121	[34]
		Waste gas emission per unit industrial added value (10,000 ton) (−)	Environmental pollution	0.0198	[35]
	Response	Domestic waste treatment rate (%) (+)	Environmental protection	0.1653	[36]
		Sewage treatment rate (%) (+)	Environmental protection	0.0834	[36]
HQED	Innovative development	The proportion of scientific research personnel expenditure in GDP (%) (+)	Innovation investment	0.0690	[37]
		Proportion of R&D expenditure to GDP (Person/10,000) (+)	Innovation investment	0.0944	[38]
		Proportion of high-tech enterprises in China (%) (+)	Innovation contribution	0.1842	[37]
	Coordinated development	Per capita GDP (RMB) (+)	Level of income coordination	0.0156	[39]
		The ratio of per capita consumption level to the national consumption level (%) (+)	Level of consumption coordination	0.0084	[40]
		Proportion of output value of secondary and tertiary industries in total output value (%) (+)	Industrial structure	0.0367	[41]
	Open development	Proportion of foreign direct investment in China (%) (+)	Openness of foreign investment	0.1443	[42]
		Proportion of total foreign trade imports and exports in the country (%) (+)	Openness of foreign trade investment	0.1571	[42]
		Proportion of inbound tourists in China (%) (+)	Openness of tourism	0.1922	[43]
	Shared development	Number of doctors per thousand (Person/thousand) (+)	Social welfare	0.0142	[44]
		Proportion of social security and employment expenditure in general public budget expenditure (%) (+)	Social security	0.0553	[45]
		Registered unemployment rate (%) (+)	Social stability	0.0287	[46]

**Table 2:** Classification of coupling degree.

Coupling Level	Dvalue Range
Advanced coupling	*C* ∈ [0.7, 1.0]
Running-in coupling	*C* ∈ [0.5, 0.7]
Antagonistic coupling	*C* ∈ [0.3, 0.5]
Low-level coupling	*C* ∈ [0.0, 0.3]

**Table 3:** Coupling degree of each city.

	2010	2011	2012	2013	2014	2015	2016	2017	2018	2019
Wuhan	0.82	0.786	0.818	0.823	0.815	0.834	0.839	0.789	0.806	0.76
Huangshi	0.927	0.984	0.99	0.981	0.983	0.985	0.994	0.989	0.935	1
Ezhou	0.986	1	0.999	0.996	0.984	0.98	0.984	0.993	1	0.995
Huanggang	0.992	0.605	0.998	0.993	0.955	0.997	0.951	0.931	0.907	0.657
Xiaogan	0.873	0.847	0.701	0.815	0.942	0.92	0.899	0.977	0.809	0.854
Xianning	0.991	0.943	0.911	0.929	0.925	0.943	0.917	0.709	0.712	0.675
Xiantao	0.606	0.59	0.876	0.803	0.756	0.857	0.805	0.793	0.772	0.779
Qianjiang	0.997	0.982	0.84	0.834	0.875	0.885	0.829	0.946	0.959	0.956
Tianmen	0.561	0.458	0.744	0.467	0.509	0.536	0.475	0.489	0.506	0.52
Xiangyang	0.846	0.837	0.868	0.881	0.935	0.919	0.907	0.906	0.865	0.869
Yichang	0.645	0.701	0.728	0.756	0.766	0.8	0.797	0.81	0.799	0.81
Jingzhou	0.477	0.511	0.543	0.629	0.539	0.57	0.538	0.656	0.597	0.706
Jingmen	0.607	0.632	0.632	0.652	0.67	0.707	0.747	0.84	0.745	0.762
Changsha	0.964	0.961	0.944	0.929	0.934	0.934	0.98	0.958	0.961	0.932
Zhuzhou	0.888	0.785	0.913	0.933	0.897	0.9	0.826	0.887	0.889	0.9
Xiangtan	0.978	0.983	0.961	0.947	0.956	0.933	0.921	0.939	0.928	0.966
Yueyang	0.76	0.82	0.889	0.885	0.922	0.952	0.864	0.91	0.888	0.907
Yiyang	0.651	0.629	0.7	0.609	0.562	0.662	0.663	0.781	0.744	0.782
Changde	0.764	0.835	0.81	0.801	0.825	0.82	0.757	0.799	0.782	0.801
Hengyang	0.89	0.967	0.963	0.954	0.945	0.962	0.916	0.941	0.898	0.914
Loudi	0.861	0.853	0.844	0.837	0.786	0.884	0.749	0.851	0.777	0.828
Nanchang	0.984	0.99	0.994	1	1	0.998	0.999	0.998	0.999	0.999
Jiujiang	0.589	0.659	0.688	0.722	0.767	0.765	0.768	0.809	0.809	0.806
Jingdezhen	0.665	0.714	0.671	0.766	0.774	0.746	0.749	0.752	0.783	0.796
Yingtan	0.813	0.837	0.801	0.864	0.867	0.835	0.87	0.986	0.862	0.772
Xinyu	0.779	0.773	0.758	0.758	0.739	0.747	0.77	0.803	0.797	0.796
Yichun	0.477	0.486	0.524	0.558	0.601	0.651	0.688	0.731	0.708	0.717
Pingxiang	0.637	0.682	0.749	0.804	0.769	0.774	0.752	0.796	0.757	0.764
Shangrao	0.533	0.584	0.607	0.635	0.679	0.693	0.798	0.822	0.802	0.782
Fuzhou	0.337	0.393	0.41	0.432	0.453	0.475	0.506	0.494	0.497	0.547
Ji’an	0.443	0.538	0.497	0.526	0.589	0.643	0.619	0.631	0.639	0.653

	Advanced coupling		*C* ∈ [0.7,1.0]			Antagonistic coupling		*C* ∈ [0.3,0.5]		
	Running in coupling		*C* ∈ [0.5,0.7]			Low level coupling		*C* ∈ [0.0,0.3]		

**Table 4:** Classification of coordination degree.

Coordination Level	Dvalue Range
High-quality coordination	*D* ∈ [0.7, 1.0]
Good coordination	*D* ∈ [0.5, 0.7]
Moderate coordination	*D* ∈ [0.3, 0.5]
Low coordination	*D* ∈ [0.0, 0.3]

**Table 5:** Coupling coordination degree of each city.

	2010	2011	2012	2013	2014	2015	2016	2017	2018	2019
Wuhan	0.596	0.619	0.612	0.623	0.632	0.649	0.655	0.628	0.652	0.675
Huangshi	0.423	0.48	0.573	0.559	0.557	0.562	0.581	0.588	0.462	0.556
Ezhou	0.458	0.49	0.511	0.515	0.49	0.496	0.507	0.533	0.565	0.602
Huanggang	0.308	0.172	0.329	0.336	0.4	0.376	0.439	0.486	0.515	0.397
Xiaogan	0.394	0.389	0.385	0.347	0.322	0.348	0.372	0.53	0.414	0.442
Xianning	0.48	0.525	0.571	0.573	0.576	0.599	0.597	0.463	0.468	0.454
Xiantao	0.267	0.293	0.255	0.293	0.324	0.3	0.322	0.335	0.363	0.366
Qianjiang	0.28	0.358	0.407	0.412	0.421	0.442	0.434	0.526	0.527	0.531
Tianmen	0.18	0.218	0.379	0.303	0.304	0.316	0.294	0.304	0.316	0.321
Xiangyang	0.458	0.468	0.496	0.513	0.5	0.534	0.534	0.576	0.553	0.567
Yichang	0.54	0.561	0.58	0.602	0.609	0.635	0.642	0.659	0.649	0.649
Jingzhou	0.268	0.275	0.291	0.288	0.305	0.303	0.33	0.379	0.366	0.416
Jingmen	0.416	0.421	0.455	0.481	0.491	0.498	0.532	0.614	0.569	0.561
Changsha	0.617	0.607	0.632	0.654	0.669	0.654	0.602	0.632	0.648	0.682
Zhuzhou	0.503	0.515	0.546	0.524	0.557	0.524	0.51	0.548	0.553	0.564
Xiangtan	0.408	0.412	0.484	0.478	0.492	0.475	0.481	0.517	0.512	0.524
Yueyang	0.456	0.487	0.527	0.522	0.536	0.525	0.469	0.529	0.505	0.526
Yiyang	0.373	0.382	0.426	0.383	0.438	0.412	0.42	0.459	0.459	0.477
Changde	0.44	0.492	0.509	0.512	0.526	0.548	0.499	0.537	0.529	0.543
Hengyang	0.36	0.322	0.392	0.405	0.395	0.382	0.396	0.473	0.458	0.47
Loudi	0.382	0.371	0.435	0.419	0.462	0.379	0.402	0.464	0.431	0.456
Nanchang	0.496	0.513	0.518	0.529	0.534	0.527	0.527	0.535	0.548	0.551
Jiujiang	0.538	0.563	0.573	0.581	0.581	0.589	0.598	0.605	0.616	0.628
Jingdezhen	0.533	0.526	0.563	0.521	0.531	0.515	0.515	0.596	0.572	0.572
Yingtan	0.49	0.493	0.539	0.53	0.535	0.541	0.553	0.656	0.499	0.525
Xinyu	0.525	0.541	0.534	0.52	0.509	0.508	0.517	0.537	0.537	0.535
Yichun	0.368	0.394	0.416	0.426	0.45	0.443	0.466	0.52	0.518	0.544
Pingxiang	0.375	0.385	0.408	0.402	0.394	0.403	0.426	0.443	0.45	0.447
Shangrao	0.434	0.456	0.468	0.475	0.491	0.497	0.474	0.528	0.523	0.531
Fuzhou	0.388	0.422	0.432	0.45	0.458	0.459	0.478	0.48	0.49	0.533
Ji’an	0.456	0.461	0.511	0.53	0.536	0.572	0.564	0.568	0.571	0.598

	High quality coordination		*D* ∈ [0.7,1.0]			Moderate coordination		*D* ∈ [0.3,0.5]		
	Good coordination		*D* ∈ [0.5,0.7]			Low coordination		*D* ∈ [0.0,0.3]		

## Data Availability

The data will be made available on request.

## References

[1] Xue A., Zhang D. (2022). Institutional Analysis of Global Economic Governance Dilemma. World Econ. Stud..

[2] Lin Z. (2018). Some Opinions on the High-Quality Development of China’s Economy. Youth J..

[3] Bonnaveira G., Paterson E. (2018). The INCN Red List of Threatened Species.

[4] Wendling Z., Emerson J., De Sherbinin A., Esty D. (2020). Environmental Per—Formance Index.

[5] Mlachila M., Tapsoba R., Tapsoba S. (2017). A Quality of Growth Index for Developing Countries: A Proposal. Soc. Indic. Res..

[6] Chen S., Chen D. (2018). Haze Pollution, Government Governance and High-Quality Economic Development. Econ. Res. J..

[7] Wei M., Li S. (2018). Construction and Measurement of the Evaluation System of China’s Economic Growth Quality under the New Normal. Economist.

[8] Zhang X., Liu X. (2021). Dialectical Thinking of Xi Jinping’s Concept of High-Quality Development in the New Era. Shandong Soc. Sci..

[9] Li Z. (2021). Motivation Mechanism for High-Quality Development of China’s Economy. Contemp. Econ. Res..

[10] Zheng W., Ou Y. (2022). High-Quality Development of Higher Education: Connotation, Challenge and Path. Mod. Educ. Manag..

[11] Li P., Deng A. (2022). Path Analysis of High-Quality Tourism Development and Common Prosperity. Soc. Sci..

[12] Ren B., Wen F. (2018). Criteria, Determinants and Ways to Achieve High-Quality Development in China in the New Era. Reform.

[13] Han L., Zhong J. (2021). Interpretation of the Connotation, Theoretical Framework and Realization Path of High-Quality Development. J. Xiangtan Univ. (Philos. Soc. Sci.).

[14] Song D., Zhang Q. (2022). The Evolution and Driving Force of the Integration of Environmental Protection and High-Quality Economic Development. J. Quant. Technol. Econ..

[15] Bradford D., Fender R., Shore S., Wagner M. (2011). The Environmental Kuznets Curve: Exploring a Fresh Specification. Contrib. Econ. Anal. PolicyCesifo Work. Pap..

[16] Panayotou T. (1995). Environment Degradation at Different Stages of Economic Development Livelihoods in the Third World.

[17] Dinda S. (2004). Environmental Kuznets Curve Hypothesis: A Survey. Ecol. Econ..

[18] Guo H., Hu C. (2022). Ecological Environmental Protection and High-Quality Development of Industrial Economy in the Yellow River Basin: Coupling Measurement and Space-Time Evolution. Ningxia Soc. Sci..

[19] Shi B., Fan D. (2022). Study on the Coupling and Coordination of Ecological Environmental Protection and High-Quality Urban Economic de-Velopment in the Northwest of the Middle and Upper Reaches of the Yellow River. Ningxia Soc. Sci..

[20] Tian Y., Lin Z. (2022). Coupling and Coordination of Agricultural Carbon Emission Efficiency and Economic Growth in China’s Provinces. Chin. J. Popul. Resour. Environ..

[21] Li X., He A. (2022). Harmonious Coexistence of Man and Nature: A Study of the Ecological Dimension of the Chinese Path to Modernization Road. Social. Stud..

[22] Ren B., He M. (2020). The Dilemma of High-Quality Development of China’s New Economy and Its Path Choice. J. Northwest Univ. (Philos. Soc. Sci. Ed.).

[23] The Development Plan of Urban Agglomeration in the Middle Reaches of the Yangtze River. https://www.ndrc.gov.cn/xxgk/zcfb/tz/201504/t20150416_963800.html.

[24] Deng C., Liang P., Liu C. (2020). Characteristics of Spatiotemporal Changes and Influencing Factors of Urban Agglomeration Contraction in the Middle Reaches of the Yangtze River. J. Urban Sci..

[25] Liu Y., Su K. (2021). Coupling and Coordinating Relationship between Tourism Economy and Ecological Environment—A Case Study of Nagasaki Prefecture, Japan. Int. J. Environ. Res. Public Health.

[26] Jian P., Jian S., Ya J. (2012). Conceptual Framework of Regional Ecological Sustainability Evaluation Based on PSR Model. Adv. Geogr. Sci..

[27] Zhang G., Wang Y., Wu K. (2020). Spatial-Temporal Characteristics and Influencing Factors of Coordination Be- Tween Economic and Environmental Development of Three Major Urban Agglomerations in China. Geogr. Res..

[28] Wu S., Liu W., Pan Y., Deng H., Jiao K., Yin Y. (2016). Range and Rate of Regional Variation of China’s Land Surface from 1960 to 2011. Chin. Sci. Bull..

[29] Zou C., Zhu J., Lou K., Yang L. (2022). Coupling Coordination and Spatiotemporal Heterogeneity between Urbanization and Ecological Environment in Shaanxi Province, China. Ecol. Indic..

[30] Zhang G., Wang Y., Wang J., Cao C. (2021). The Spatiotemporal Evolution and Mechanism of the Coordinated Relationship between the Quality and Quantity of Economic Growth in China’s Urban Agglomeration. Sci. Geogr. Sin..

[31] Hu X., Xu P. (2020). Research on the Impact of FDI Quality Characteristics on the High-Quality Development of China’s Economy. J. Int. Trade.

[32] Li Z., Chen Y., Liu Y. (2022). Multidimensional Performance of High-Quality Development from the Perspective of “Governance of China. ” Reform.

[33] Hou X., Zhu Q., Wan C. (2022). Centennial Common Prosperity: Evolution, Theoretical Innovation and Path Choice. Econ. Probl..

[34] Lei D., Xu X., Zhang Y. (2021). Analysis of the Dynamic Characteristics of the Coupling Relationship between Urbanization and Environment in Kun-Ming City, Southwest China. Clean. Environ. Syst..

[35] Shi T., Yang S., Zhang W., Zhou Q. (2020). Coupling Coordination Degree Measurement and Spatiotemporal Heterogeneity between Economic Development and Ecological Environment ––Empirical Evidence from Tropical and Subtropical Regions of China. J. Clean. Prod..

[36] Luo L., Wang Y., Liu Y., Zhang X., Fang X. (2022). Where Is the Pathway to Sustainable Urban Development? Coupling Coordination Evaluation and Configuration Analysis between Low-Carbon Development and Eco-Environment: A Case Study of the Yellow River Basin, China. Ecol. Indic..

[37] Yang G., Gong G., Luo Y., Yang Y., Gui Q. (2022). Spatiotemporal Characteristics and Influencing Factors of Tourism–Urbanization–Technology–Ecological Environment on the Yunnan–Guizhou–Sichuan Region: An Uncoordinated Coupling Perspective. Int. J. Environ. Res. Public Health.

[38] Min L., Xu Z. (2022). Digital Economy, Innovation Performance and High-Quality Economic Development—Based on Empirical Evidence of Chinese Cities. Stat. Decis..

[39] Xiaomin G., Chuanglin F., Xufang M., Dan C. (2022). Coupling and Coordination Analysis of Urbanization and Ecosystem Service Value in Beijing-Tianjin-Hebei Urban Agglomeration. Ecol. Indic..

[40] Wu X., Zhang Y., Wang L. (2022). Coupling Relationship between Regional Urban Development and Eco-Environment: Inspiration from the Old Industrial Base in Northeast China. Ecol. Indic..

[41] Li W., Cai Z., Jin L. (2022). Spatiotemporal Characteristics and Influencing Factors of the Coupling Coordinated Development of Production-Living-Ecology System in China. Ecol. Indic..

[42] Ge D., Chen Q., Lai Z. (2021). Analysis on the Coupling and Coordination of Tourism, Economy and Ecological Environment in China’s Provinces. Ecol. Econ..

[43] Ma M., Tang J. (2022). Interactive Coercive Relationship and Spatio-Temporal Coupling Coordination Degree between Tourism Urbanization and Eco-Environment: A Case Study in Western China. Ecol. Indic..

[44] Han W., Chen X., Pang J., Wang N., Yu Y. (2018). Study on the Coupling and Coordinated Development of Urbanization, Ecological Environment and Tourism Industry—Taking 9 Provinces (Districts and Cities) of the Silk Road Economic Belt as an Example. J. Lanzhou Univ. (Nat. Sci. Ed.).

[45] Huang H. (2020). Research on the Quality Development of New Urbanization with Chinese Characteristics. China Stat..

[46] Xiao Y., Tang X., Wang J., Huang H., Liu L. (2022). Assessment of Coordinated Development between Tourism Development and Resource Environment Carrying Capacity: A Case Study of Yangtze River Economic Belt in China. Ecol. Indic..

[47] Li Q., Zhao Y., Li S., Li X. (2020). Analysis of the Spatial and Temporal Characteristics and Driving Forces of the Coupling of Social Security and Economic Development in China. Geogr. Res..

[48] Meng Q., Du H., Wang J. (2017). Analysis of Local Government Haze Governance Behavior from the Perspective of Interest Appeal. China Soft Sci..

[49] Zhai T., Wang J., Jin Z., Qi Y. (2019). Analysis on the Change and Correlation of the Supply and Demand Pattern of Ecosystem Services in the Yangtze River Economic Belt. Acta Ecol. Sin..

[50] Huang H., Hu Q., Qiao X. (2018). Research on Dynamic Changes of Ecological Efficiency in Jiangxi Province Based on Green GDP and Ecological Footprint. Acta Ecol. Sin..

[51] Hu X., Zou L. (2020). Study on the Evolution and Driving Mechanism of Land Use Landscape Pattern in the Yangtze River Economic Belt under the Guidance of Ecological Priority—Taking Wuhan as an Example. Areal Res. Dev..

[52] Zhu Z., He Q. (2016). Dynamic Simulation of Urban Spatial Structure Evolution in Changsha. Econ. Geogr..

[53] Zhao X. (2015). Realize Industrial Integration of the Yangtze River Economic Belt Relying on Urban Agglomeration—Take the Automo-Bile Industry of Urban Agglomeration in the Middle Reaches of the Yangtze River as an Example. China Dev..

[54] Yu Y., Wang C., Peng L., Yu Y. (2022). Evaluation of Agricultural Green Development Level and Analysis of Obstacles Based on Entropy Weight TOPSIS Model—Taking Jiangxi Province as an Example. Chin. J. Agric. Resour. Reg. Plan..

[55] 2019 Statistical Yearbook. https://tjj.hubei.gov.cn/tjsj/sjkscx/tjnj/qstjnj/.

[56] Wang J. (2021). The Centennial Course of China’s Regional Economic Development—Based on the Relationship between Rationality and Efficiency. Contemp. Econ. Manag..

[57] Li B. (2022). Calculation and Regional Difference Comparison of the Equalization Level of Basic Public Services in Urban Agglomerations in the Middle Reaches of the Yangtze River. Stat. Decis..

[58] Zhang S. (2019). Empirical Analysis of the Relationship between Economic Growth and Environmental Quality Based on Kuznets Model—Taking Anhui Province as an Example. J. Heilongjiang Univ. Technol. (Compr. Ed.).

[59] Yang Z., Liu Y., He Y. (2007). Discussion on the Establishment of Eco-Friendly Land Use Strategy in China. Resour. Sci..

